# Regulation of Heat Shock Factor Pathways by γ-aminobutyric Acid (GABA) Associated with Thermotolerance of Creeping Bentgrass

**DOI:** 10.3390/ijms20194713

**Published:** 2019-09-23

**Authors:** Ting Liu, Zhaoqiao Liu, Zhou Li, Yan Peng, Xinquan Zhang, Xiao Ma, Linkai Huang, Wei Liu, Gang Nie, Liwen He

**Affiliations:** Department of Grassland Science, College of Animal Science and Technology, Sichuan Agricultural University, Chengdu 611130, China; liutin_cn@163.com (T.L.); Liuzq9833@163.com (Z.L.); pengyanlee@163.com (Y.P.); zhangxq@sicau.edu.cn (X.Z.); maroar@126.com (X.M.); huanglinkai@sicau.edu.cn (L.H.); lwgrass@126.com (W.L.); nieg17@sicau.edu.cn (G.N.); shenjiu726@163.com (L.H.)

**Keywords:** antioxidant defense, gene expression, heat shock protein, heat shock factor, oxidative damage, photosynthesis

## Abstract

Activation and enhancement of heat shock factor (HSF) pathways are important adaptive responses to heat stress in plants. The γ-aminobutyric acid (GABA) plays an important role in regulating heat tolerance, but it is unclear whether GABA-induced thermotolerance is associated with activation of HSF pathways in plants. In this study, the changes of endogenous GABA level affecting physiological responses and genes involved in HSF pathways were investigated in creeping bentgrass during heat stress. The increase in endogenous GABA content induced by exogenous application of GABA effectively alleviated heat damage, as reflected by higher leaf relative water content, cell membrane stability, photosynthesis, and lower oxidative damage. Contrarily, the inhibition of GABA accumulation by the application of GABA biosynthesis inhibitor further aggravated heat damage. Transcriptional analyses showed that exogenous GABA could significantly upregulate transcript levels of genes encoding heat shock factor HSFs (*HSFA-6a*, *HSFA-2c*, and *HSFB-2b*), heat shock proteins (*HSP17.8*, *HSP26.7*, *HSP70*, and *HSP90.1-b1*), and ascorbate peroxidase 3 (*APX3*), whereas the inhibition of GABA biosynthesis depressed these genes expression under heat stress. Our results indicate GABA regulates thermotolerance associated with activation and enhancement of HSF pathways in creeping bentgrass.

## 1. Introduction

Persistent high temperature in summer seriously affects the normal growth and development of plants. Heat stress has become one of the main abiotic stresses limiting crop production and the quality of ground cover plants due to the aggravation of global warming [1,2]. Cool-season turfgrasses such as creeping bentgrass (*Agrostis stolonifera* L.) adapt to cool-humid and cool-arid climates, but are susceptible to heat stress [3,4]. However, creeping bentgrass has become the first choice for establishing high-quality turf, such as the putting green in a golf course, because of its fine texture, charming color, and stoloniferous habit [4,5]. It is of great significance to find an effective way to improve heat tolerance of creeping bentgrass in summer. The application of plant growth regulators (PGRs) for improving heat tolerance of plants has many advantages including low cost and environmental risk, simple operation, and stable effect [6]. Previous studies have shown that suitable dose of exogenous PGRs such as fructose, abscisic acid (ABA), and salicylic acid (SA) can significantly improve heat tolerance of creeping bentgrass [7,8].

γ-Aminobutyric acid (GABA) is a non-protein amino acid and widely exists in eukaryotes and prokaryotes [9]. As an important nerve conduction signal in animals, GABA has been broadly studied and its function is involved in metabolic regulation, antioxidation, and nutrient digestibility under stress condition [10,11]. In higher plants, GABA acts as an important nitrogen source and intermediator of metabolism for plant growth and development and also quickly accumulates in response to various environmental stresses including high temperature stress [12,13,14]. Previous studies have confirmed that GABA priming effectively enhanced tolerance to osmotic stress in black pepper (*Piper nigrum*) associated with osmotic adjustment and antioxidant defense [15]. GABA treatment could alleviate chilling injury of *Anthurium* spp. cut flowers by enhancing GABA shunt, ATP supply, and endogenous glycine betaine accumulation [16]. Exogenous application of GABA significantly increased the growth and survival rate of four-day-old rice (*Oryza sativa*) under heat stress by improving leaf turgor and upregulating osmoprotectants and antioxidants [17]. GABA may act as a signal molecule interacting with plant hormones and other metabolites, which regulates heat tolerance in *Arabidopsis thaliana* [18]. Foliar application of GABA induced heat tolerance of creeping bentgrass involved in regulating osmotic potential, tricarboxylic acid cycle, and metabolic homeostasis [14]. However, limited researches reveal possible effects of GABA on regulating heat tolerance in plants at the transcriptional level.

When plants are exposed to a high-temperature environment, their normal growth and development will be inhibited and physiological metabolism will be disordered [14,19]. A typical heat-induced symptom is leaves turning yellow and withered due to oxidative damage, chlorophyll degradation, and water imbalance [20,21]. Early sensing and transduction of heat stress signals in plants are mainly mediated by heat shock factors (HSFs) leading to the production of various cell-protective molecular chaperone proteins such as heat shock proteins (HSPs) [22]. Activation and enhancement of HSF pathways are important adaptive responses to heat stress in plants. HSFs are critical transcription factors involved in transcriptional activation of heat shock genes. Many downstream heat-defensive genes, including HSPs and ascorbate peroxidases (APXs), could be regulated by HSFs [23,24]. HSPs function to stabilize proteins and refold stress-damaged proteins during heat stress or other abiotic stress [25,26]. APXs play important roles in scavenging stress-induced H_2_O_2_ overaccumulation, thereby alleviating oxidative damage in plants [27]. However, it still needs to be further studied whether the mediation of endogenous GABA level affects HSFs pathways contributing to heat tolerance in plants. Objectives of this study were to investigate effects of GABA and its biosynthetic inhibitor 3-mercaptopropionic acid (3-MPA) on physiological changes including water status, photosynthesis, oxidative damage, and antioxidant capacity in creeping bentgrass during a prolonged period of high temperature stress, and to further examine whether the GABA-induced heat tolerance is related to the activation and enhancement of HSF pathways. The current study will contribute to better understanding of the positive function of GABA on regulating heat tolerance in plants at the transcriptional level.

## 2. Results 

### 2.1. The Mediation of Endogenous GABA Level Affects Water Status and Photosynthesis during Heat Stress

**Figure 1:**
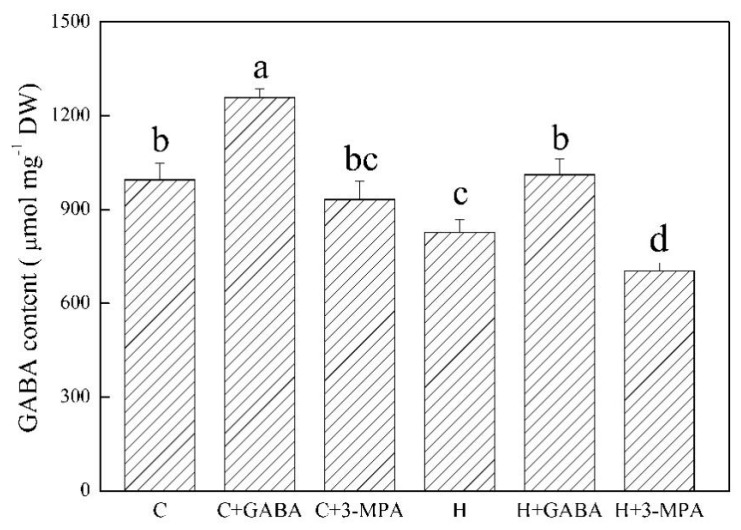
Effects of γ-aminobutyric acid (GABA) and 3-mercaptopropionic acid (3-MPA) on endogenous GABA content in leaf of creeping bentgrass at 30 d of 23/18 (normal condition) or 38/33 °C (heat stress). Vertical bars above columns indicate ±standard error (SE) of means (*n* = 4) and different letters above columns indicate significant differences (*p* ≤ 0.05). C: control; C+GABA: control+GABA; C+3-MPA: control+3-MPA; H: heat; H+GABA: heat+GABA; H+3-MPA: heat+3-MPA.

**Figure 2:**
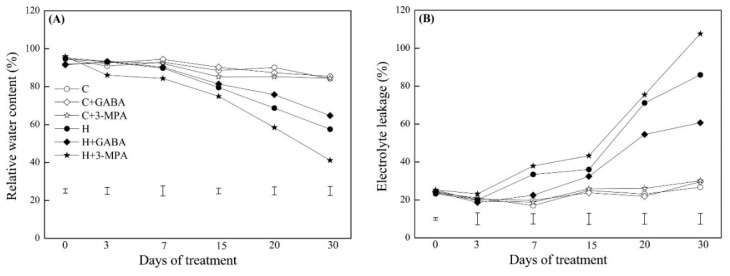
Effects of γ-aminobutyric acid (GABA) and 3-mercaptopropionic acid (3-MPA) on (**A**) relative water content (RWC) and (**B**) electrolyte leakage (EL) in leaf of creeping bentgrass during 30 days of 23/18 (normal condition) or 38/33 °C (heat stress). Vertical bars below curves represent least significance difference (LSD) values at a given day of treatment (*p* ≤ 0.05). C: control; C+GABA: control+GABA; C+3-MPA: control+3-MPA; H: heat; H+GABA: heat+GABA; H+3-MPA: heat+3-MPA.

**Figure 3:**
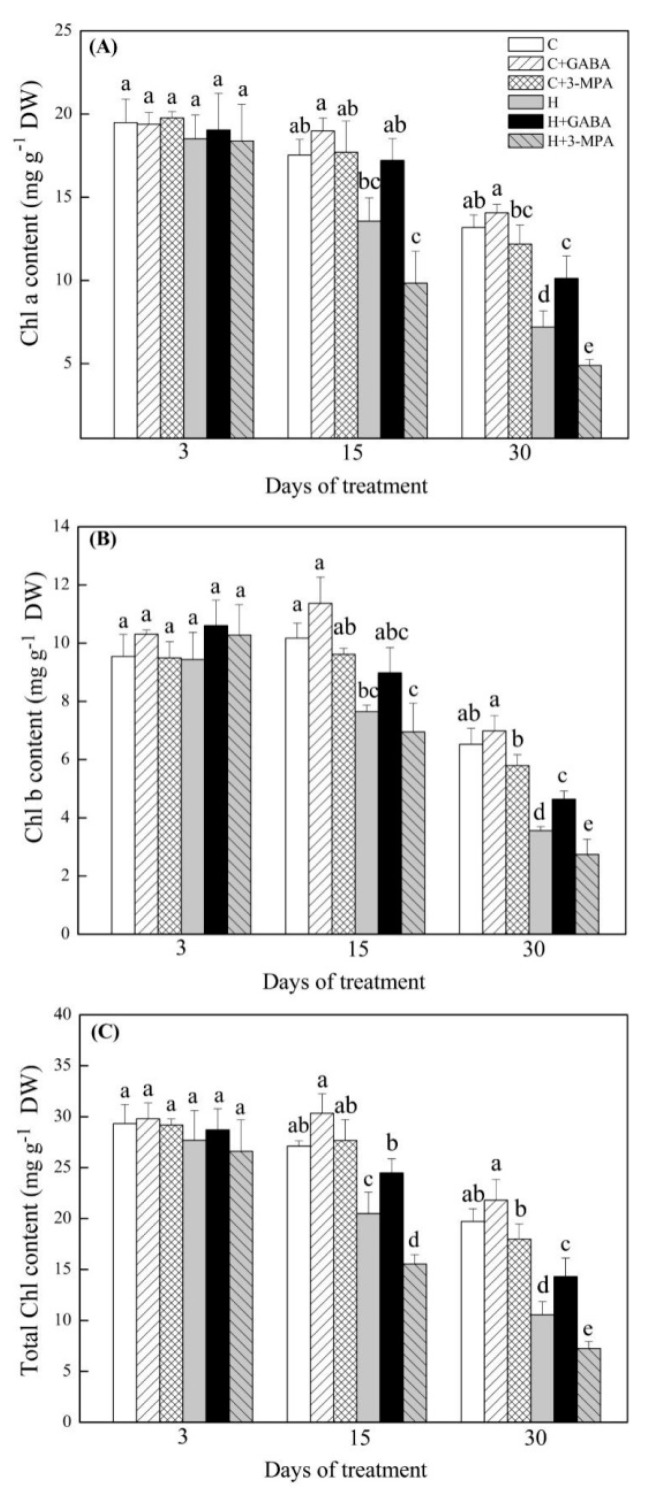
Effects of γ-aminobutyric acid (GABA) and 3-mercaptopropionic acid (3-MPA) on (**A**) chlorophyll (Chl) a content, (**B**) Chl b content, and (**C**) total Chl content in leaf of creeping bentgrass during 30 days of 23/18 (normal condition) or 38/33 °C (heat stress). Vertical bars above columns indicate ±standard error (SE) of means (*n* = 4) and different letters above columns indicate significant differences (*p* ≤ 0.05). C: control; C+GABA: control + GABA; C+3-MPA: control+3-MPA; H: heat; H+GABA: heat+GABA; H+3-MPA: heat+3-MPA.

**Figure 4:**
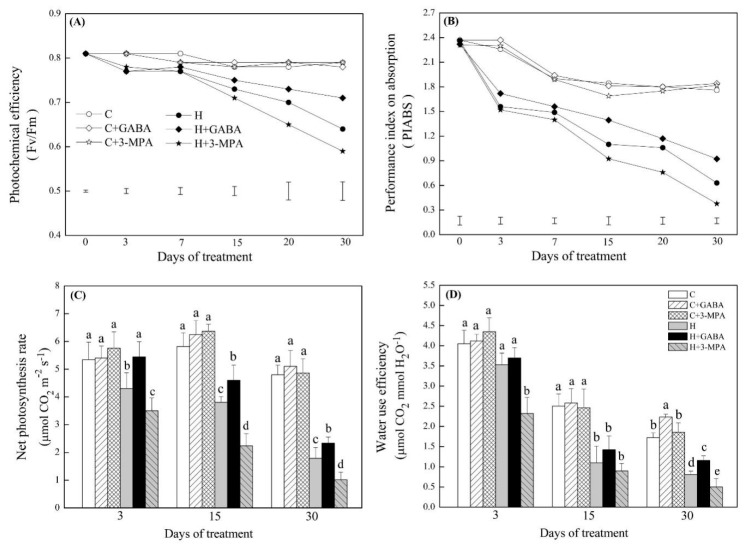
Effects of γ-aminobutyric acid (GABA) and 3-mercaptopropionic acid (3-MPA) on (**A**) photochemical efficiency (Fv/Fm), (**B**) performance index on absorption (PIABS), (**C**) net photosynthesis rate (Pn), and (**D**) water use efficiency (WUE) in leaf of creeping bentgrass during 30 days of 23/18 (normal condition) or 38/33 °C (heat stress). Vertical bars below curves represent least significance difference (LSD) values at a given day of treatment (*p* ≤ 0.05); vertical bars above columns indicate ±standard error (SE) of means (*n* = 4) and different letters above columns indicate significant differences (*p* ≤ 0.05). C: control; C+GABA: control+GABA; C+3-MPA: control+3-MPA; H: heat; H+GABA: heat + GABA; H+3-MPA: heat+3-MPA.

The GABA-pretreated creeping bentgrass (C+GABA) showed significantly higher endogenous GABA content than untreated plants (C) under normal condition. Under heat stress, heat-stressed plants with GABA pretreatment (H+GABA) had a 22% increase in GABA content than heat-stressed plants without GABA application (H). On the contrary, heat-stressed plants pretreated with 3-MPA (H+3-MPA) showed 15% decrease in GABA content than heat-stressed plants without GABA treatment (H) in leaves (Figure 1). The GABA and 3-MPA had no significant effects on relative water content (RWC) and electrolyte leakage (EL) during 30 d of the non-stress condition (Figure 2). Heat stress resulted in a significant decrease in RWC and increase in EL in untreated, GABA-treated, or 3-MPA-treated plants. The GABA-treated creeping bentgrass maintained significantly higher leaf RWC than untreated and 3-MPA-treated plants during 30 d of heat stress (Figure 2A). The exogenous application of GABA reduced heat-induced increase in EL, whereas the 3-MPA application aggravated the heat-induced increase in EL and decrease in RWC (Figure 2). Compared to the untreated control, exogenous GABA and 3-MPA had no significant effects on chlorophyll (Chl) content in plants under normal condition, but GABA-treated plants showed significantly higher total Chl than 3-MPA-treated plants at 30 d of normal condition (Figure 3). Under heat stress, GABA-treated plants maintained higher Chl content than untreated and 3-MPA-treated plants, while the 3-MPA-treated plants had the lower Chl content than GABA-treated and untreated plants in leaves. There were significant differences in Chl a, Chl b, and total Chl among three treatments (H, H+GABA, and H+3-MPA) after 30 days of heat stress (Figure 3). For changes of photosynthesis and water use efficiency (WUE), exogenous GABA and 3-MPA had no significant effects on photochemical efficiency (Fv/Fm), performance index on absorption (PIABS), and net photosynthetic rate (Pn), but the GABA application obviously improved WUE in leaves under normal condition (Figure 4). Heat stress caused persistent declines in Pn, Fv/Fm, PIABS, and WUE in all treatments. The application of GABA or 3-MPA alleviated or aggravated heat-induced decreases in Fv/Fm, PIABS, Pn, and WUE when plants were subjected to 30 d of heat stress. The heat-stressed plants with GABA pretreatment exhibited a 30%, 11%, 46%, or 43% increase in Pn, Fv/Fm, and PIABS, or WUE than heat-stressed plants without GABA pretreatment, respectively. On the contrary, the Chl, Pn, Fv/Fm, or PIABS was significantly lower in heat-stressed plants pretreated with 3-MPA than that in heat-stressed plants without 3-MPA treatment, respectively (Figure 4).

### 2.2. The Mediation of Endogenous GABA Level Affects Antioxidant Capacity and Oxidative Damage under Heat Stress

**Figure 5:**
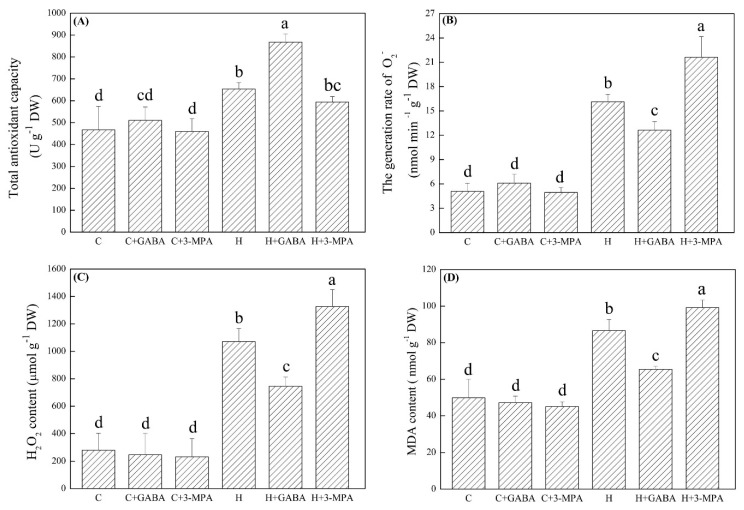
Effects of γ-aminobutyric acid (GABA) and 3-mercaptopropionic acid (3-MPA) on (**A**) total antioxidant capacity (TAC), (**B**) superoxide anion (O_2_.^−^), (**C**) hydrogen peroxide (H_2_O_2_), and (**D**) malondialdehyde (MDA) content in leaf of creeping bentgrass at 30 d of 23/18 (normal condition) or 38/33 °C (heat stress). Vertical bars above columns indicate ±standard error (SE) of means (*n* = 4) and different letters above columns indicate significant differences (*p* ≤ 0.05). C: control; C+GABA: control + GABA; C+3-MPA: control+3-MPA; H: heat; H+GABA: heat + GABA; H+3-MPA: heat+3-MPA.

The total antioxidant capacity (TAC), generation of superoxide anion (O_2_^−^), hydrogen peroxide (H_2_O_2_), and malondialdehyde (MDA) content were not significantly affected by exogenous application of GABA and 3-MPA under normal condition (Figure 5). The TAC in GABA-treated and untreated plants (H+GABA and H) were obviously improved by heat stress. GABA-treated plants exhibited 33% increase in TAC than untreated plants under heat stress. On the contrary, the TAC in heat-stressed plants with 3-MPA pretreatment (H+3-MPA) decreased by 9%, as compared to heat-stressed plants without 3-MPA treatment (H) (Figure 5A). As shown in Figure 5B–D, heat stress significantly induced the accumulation of O_2_.^−^, H_2_O_2_, and MDA in all plants. However, exogenous GABA or 3-MPA decreased or enhanced heat-induced O_2_.^−^, H_2_O_2_, and MDA accumulation in leaves (Figure 5B–D). The generation rate of O_2_.^−^ in GABA-pretreated plants decreased by 22% than that in untreated plants under heat stress (Figure 5B). Similarly, GABA-pretreated plants showed 30% and 24% decreases in H_2_O_2_ and MDA content than untreated plants in response to heat stress, respectively (Figure 5C,D). The O_2_.^−^, H_2_O_2_, and MDA of 3-MPA-pretreated plants were 34%, 24%, and 14% higher than those of untreated plants under heat stress, respectively (Figure 5A–D).

### 2.3. The Mediation of Endogenous GABA Level Affects HSFs Pathways during Heat Stress

**Figure 6:**
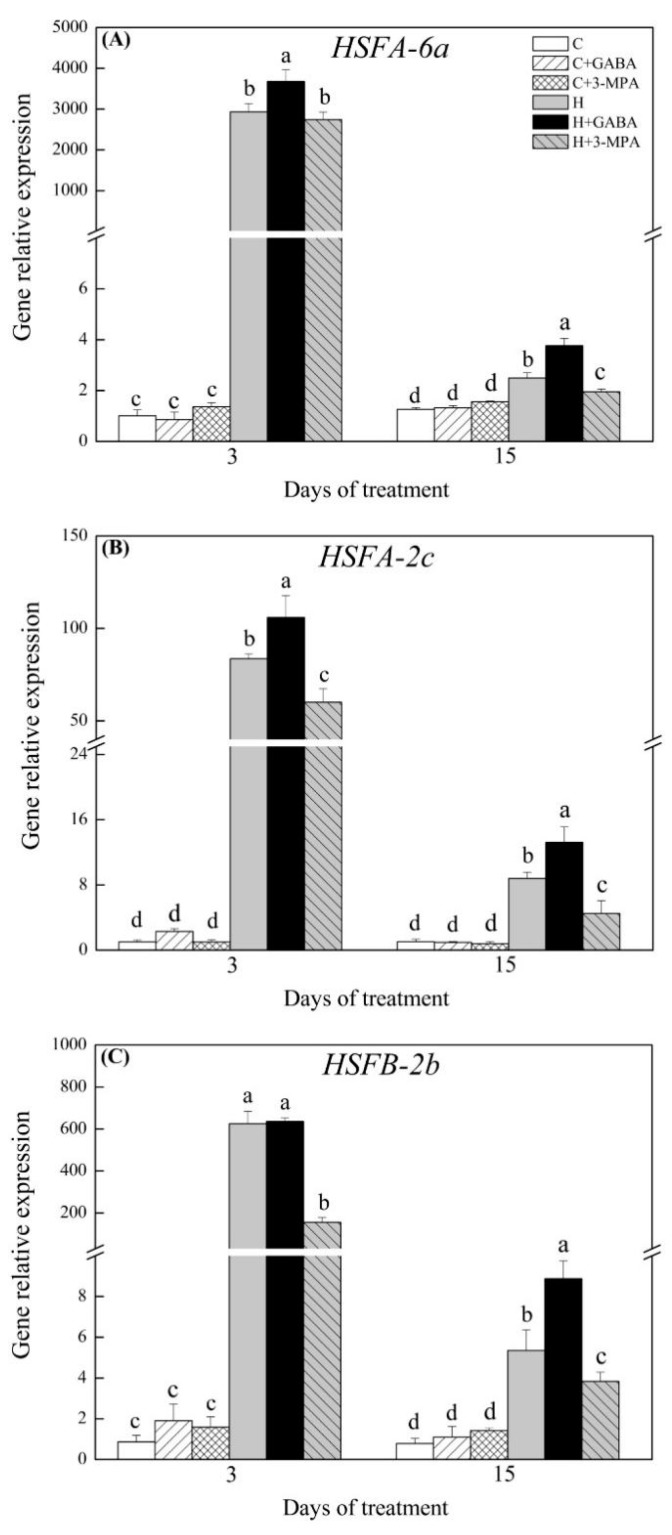
Effects of γ-aminobutyric acid (GABA) and 3-mercaptopropionic acid (3-MPA) on (**A**) *HSFA-2a*, (**B**) *HSFA-2c*, and (**C**) *HSFB-2b* relative expression in leaves of creeping bentgrass at 3 and 15 d of 23/18 (normal condition) or 38/33 °C (heat stress). Vertical bars above columns indicate ±standard error (SE) of means (*n* = 4) and different letters above columns indicate significant differences (*p* ≤ 0.05). C: control; C+GABA: control + GABA; C+3-MPA: control+3-MPA; H: heat; H+GABA: heat + GABA; H+3-MPA: heat+3-MPA.

**Figure 7:**
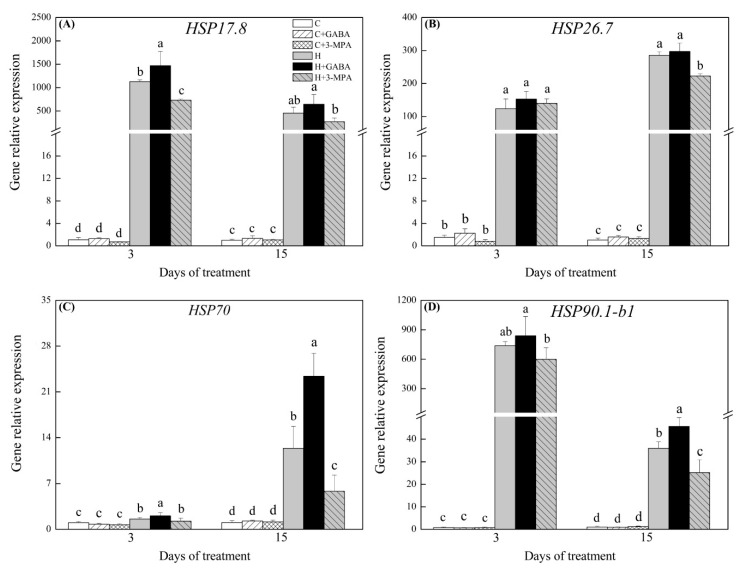
Effects of γ-aminobutyric acid (GABA) and 3-mercaptopropionic acid (3-MPA) on (**A**) *HSP17.8* (**B**) *HSP26.7*, (**C**) *HSP70*, and (**D**) *HSP90.1-b1* relative expression in leaves of creeping bentgrass at 3 and 15 d of 23/18 (normal condition) or 38/33 °C (heat stress). Vertical bars above columns indicate ±standard error (SE) of means (*n* = 4) and different letters above columns indicate significant differences (*p* ≤ 0.05). C: control; C+GABA: control + GABA; C+3-MPA: control+3-MPA; H: heat; H+GABA: heat+GABA; H+3-MPA: heat+3-MPA.

**Figure 8:**
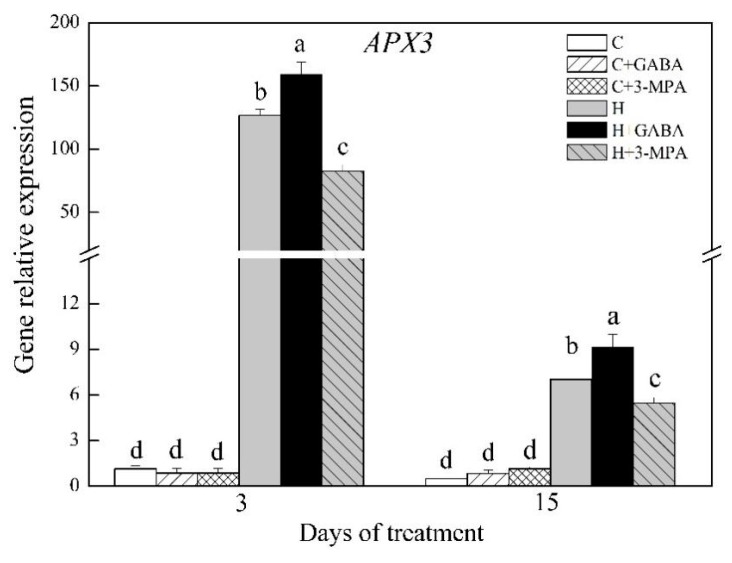
Effects of γ-aminobutyric acid (GABA) and 3-mercaptopropionic acid (3-MPA) on *APX3* relative expression in leaves of creeping bentgrass at 3 and 15 d of 23/18 (normal condition) or 38/33 °C (heat stress). Vertical bars above columns indicate ±standard error (SE) of means (*n* = 4) and different letters above columns indicate significant differences (*p* ≤ 0.05). C: control; C+GABA: control + GABA; C+3-MPA: control+3-MPA; H: heat; H+GABA: heat+GABA; H+3-MPA: heat+3-MPA.

**Figure 9:**
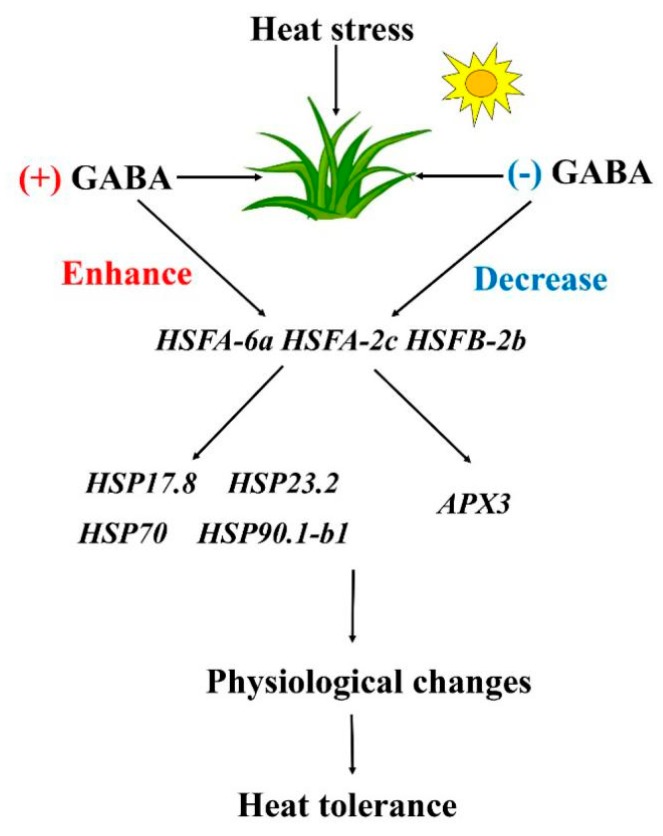
Proposed heat shock factor pathways regulated by γ-aminobutyric acid (GABA) in creeping bentgrass under heat stress.

Key genes involved in HSFs pathways were analyzed, including *HSFs* (*HSFA-6a*, *HSFA-2c*, and *HSFB-2b*), *HSPs* (*HSP17.8*, *HSP26.7*, *HSP70*, and *HSP90.1-b1*), and *APX3* under normal and heat stress conditions (Figure 6, Figure 7 and Figure 8). Under normal condition, exogenous GABA and 3-MPA had no significant influences on gene expression levels of HSFs pathways. Heat stress significantly upregulated all genes involved in HSFs pathways in spite of GABA and 3-MPA pretreatment. Obviously, exogenous application of GABA further upregulated heat-induced expression of *HSFA-6a*, *HSFA-2c*, and *HSFB-2b*, whereas the 3-MPA application remarkably inhibited heat-induced expression of these genes. The GABA-treated plants had 51%, 50%, and 66% higher transcript levels of *HSFA-6a*, *HSFA-2c*, or *HSFB-2b*, respectively, than untreated plants at 15 d of heat stress (Figure 6A–C). When creeping bentgrass was subjected to 3 d of heat stress, transcript level of *HSFA-6a* and *HSFA-2c* in 3-MPA-treated plants was downregulated by 25% and 27% than that in untreated plants, respectively (Figure 6A,B). The 3-MPA-treated plants also exhibited 75% decrease in *HSFB-2b* expression than GABA-treated and untreated plants at 3 d of heat stress (Figure 6C). It was remarkably different in *HSP17.8* expression level among untreated, GABA-treated, and 3-MPA-treated plants at 3 d of heat stress (Figure 7A). The lowest or highest *HSP17.8* expression level was found in 3-MPA-treated or GABA-treated plants at 3 d of heat stress, respectively. When the duration of heat stress reached to 15 d, the GABA-treated plants had 81% increase in *HSP17.8* expression relative to 3-MPA-treated plants (Figure 7A). The transcript level of *HSP26.7* was significantly inhibited by exogenous 3-MPA at 15 d of heat stress (Figure 7B). The GABA application further improved heat-induced increase in *HSP70* expression at 3 d of heat stress (Figure 7C). The GABA-treated or 3-MPA-treated plants had 89% or 53% increase or decrease in *HSP70* expression level than untreated plants at 15 d of heat stress, respectively (Figure 7C). Similarly, the expression level of *HSP90.1-b1* was upregulated by 27% in GABA-treated plants and downgraded by 30% in 3-MPA-treated plants as compared to that in untreated plants at 15 d of heat stress (Figure 7D). The *APX3* expression level was markedly upregulated by GABA pretreatment and dramatically downgraded by the 3-MPA under heat stress. Compared with that in untreated control, the *APX3* expression level increased by 25% or 30% in GABA-treated plants and decreased by 35% or 22% in 3-MPA-treated plants at 3 or 15 d of heat stress, respectively (Figure 8). Figure 9 shows proposed HSF pathways regulated by GABA in leaf of creeping bentgrass under heat stress.

## 3. Discussion

It is generally known that heat stress can reduce photosynthesis and cell membrane stability, accelerate leaf senescence, cause water loss, and inhibit normal growth and development of plants [28,29]. Previous studies have shown that the application of GABA can effectively improve the stress tolerance of various plant species under different stress conditions such as the tolerance to water deficit in black cumin (*Nigella sativa*) [30], the tolerance to hypoxic stress in melon (*Cucumis melo*) seedlings [31], and salt tolerance in cucumber (*Cucumis sativus*) [32]. Our present results showed that the increase in endogenous GABA content by exogenous GABA pretreatment could significantly alleviate heat-induced water loss and membrane damage in leaves of creeping bentgrass, which is consistent with previous report about effects of GABA in rice in response to high temperature stress [17]. In addition, the creeping bentgrass pretreated with GABA showed obviously higher Chl content, Fv/Fm, PIABS, Pn, and WUE than untreated plants under heat stress. In contrast, the decline in endogenous GABA content by 3-MPA application further weakened membrane stability and photosynthesis in creeping bentgrass under heat stress. These results imply that GABA-regulated heat tolerance is closely associated with the photosynthetic maintenance and delaying senescence in creeping bentgrass during heat stress. Earlier studies also found that exogenous application of GABA could effectively improve photosynthesis in pepper (*Capsicum annuum*) seedlings under low light stress, in pakchoi (*Brassica campestris*) under waterlogging stress, and in maize seedlings under salt stress [33,34,35]. The increase in WUE induced by GABA helped to reduce water loss and improve water balance when creeping bentgrass suffered from heat damage.

Plants develop very complex regulatory networks to adapt to thermal environments. Different members of HSFs participate in different regulatory processes of the heat stress response (HSR), which endows plants with heat tolerance [36,37]. In higher plants, HSPs could be divided into three categories and several subcategories according to structural characteristics and oligomerization DNA domain. There are three main types of HSFs in plants including class A members: HSF activator (HSFA) containing transcriptional activation domain AHA (aromatic, hydrophobic, and acidic amino acid residues), and class B (HSFB) and C (HSFC) without the AHA domain [37,38,39]. A previous study showed that the overexpression of *Fahsfa2b* and *Fahsfa2c* could significantly improve the heat tolerance of Arabidopsis and tall fescue (*Festuca arundinacea*) through activating downstream small *Hsps*, *Hsp70*, and *APX2* [36]. During the seed germination and seedling stage, Arabidopsis overexpressing *HsfA6a* was more tolerant to salt and drought stress than wild type, and microarray and qRT-PCR analysis showed that many stress response genes were upregulated in transgenic plants [40]. The study of Ma et al. (2016) also found that the overexpression of *CarHSFB2* enhanced tolerance against drought and heat stress in Arabidopsis and transcript levels of *HsfA2*, *HsfB2a*, and *HsfA7a* in transgenic plants were markedly higher than that in wild type in response to heat stress [41]. Our current transcriptional analyses showed that exogenous GABA could significantly upregulate transcript levels of *HSFA-6a*, *HSFA-2c*, and *HSFB-2b*, whereas the inhibition of GABA biosynthesis depressed these genes expression under heat stress. These results imply that GABA-induced heat tolerance involves the activation of *HSFA-6a*, *HSFA-2c*, and *HSFB-2b* in creeping bentgrass.

HSPs are important downstream functional genes that are regulated by HSFs [42]. As molecular chaperones, HSPs protect plants from stress damage through stabilizing proteins and ensuring proper folding and assemble of proteins. HSPs are not only induced by short-term stress shock, but also essential for heat adaptation to long-term stress [43]. It has been found that HSPs are quickly synthesized and accumulated when plants cope with high-temperature stress, which is positively correlated with heat tolerance in plants [44,45,46]. Previous study found that the heat-tolerant rice cultivar ‘Co39′ had significantly higher expression levels of small HSPs (*Hsp16.9A*, *Hsp17.4*, *Hsp17.9A*, and *Hsp23.2*) than the heat-susceptible rice cultivar “Azucena” in response to heat stress [47]. Enhanced tolerance against heat stress could be acquired by an *OsHSP26* overexpression in tall fescue [48]. Appropriate dose of nitrogen could significantly improve thermotolerance of creeping bentgrass associated with increases in HSP70 and HSP90 accumulation in leaf [49]. Overexpression of five HSP90 genes (*GmHsp90A2*, *GmHsp90A4*, *GmHsp90B1*, *GmHsp90C1.1*, and *GmHsp90C2*.1) cloning from soybean (*Gycin emax*) remarkably increased thermal protection in Arabidopsis [50]. Current findings showed that the GABA could further improved heat-upregulated transcript levels of *HSP17.8*, *HSP26.7*, *HSP70*, and *HSP90.1-b1*, whereas the inhibition of GABA biosynthesis depressed these genes expression under heat stress. Current results indicate GABA regulates thermotolerance in relation to activation and upregulation of *HSPs* which play protective roles in stabilizing proteins when creeping bentgrass responds to heat stress.

Environmental stress can significantly increase the production of reactive oxygen species (ROS) causing oxidative damage to plants. ROS can damage lipids, nucleic acids and proteins, leading to disruption of physiological processes [51]. Generally, ROS such as hydrogen peroxide (H_2_O_2_) and superoxide anion (O_2_.^−^) are maintained at lower level under normal condition, but heat stress destroys dynamic balance of ROS resulting in their overaccumulation in plant cells [19]. However, plants have developed antioxidant mechanism for ROS scavenging, which plays a critical role in promoting plants adaptation to various environmental stresses. APXs are important downstream functional genes of HSFs pathways and play important roles in antioxidant metabolism in plants [38]. It is well known that superoxide dismutase (SOD) catalyzes the disproportionation of O_2_.^−^ to H_2_O_2_ [27]. APX is a key enzyme involved in the ascorbic acid–glutathione (ASA–GSH) cycle and catalyzes the conversion of H_2_O_2_ to H_2_O and O_2_ using ASA-specific electronic donors [52]. Salt stress, drought, high or low temperature, strong light, and other environmental stimuli could induce *APXs* expression in plants [53,54,55,56,57]. It has been proved that overexpression of an Arabidopsis *APX3* in tobacco (*Nicotiana tabacum*) effectively protected plants from oxidative stress [58]. Transgenic tobacco overexpressing Arabidopsis *APX3* could also effectively alleviate drought-induced oxidative damage [59]. Photosystem II complexes (Φ_PSII_) decreased earlier in the *APX3* knockout Arabidopsis mutant compared with the wild type in response to heat stress [60]. The study of Shi et al. (2001) also found that overexpression of an *APX* (72.1% homology to the Arabidopsis *APX3*) cloning from barley (*Hordeum vulgare*) significantly enhanced thermotolerance of Arabidopsis [53]. In this study, the increase or decrease in endogenous GABA level could enhance or inhibit heat-induced *APX3* expression. Physiological analysis also proved GABA or 3-MPA effectively alleviated or aggravated heat-induced H_2_O_2_ accumulation and oxidative damage in leaves of creeping bentgrass. Thus, the GABA increased *APX3* expression resulting in enhanced H_2_O_2_ scavenging ability, which effectively alleviated heat-induced oxidative damage. Current results indicate that GABA-regulated antioxidant balance largely contributes to heat tolerance in creeping bentgrass.

## 4. Materials and Methods

### 4.1. Plant Materials and Treatments

Creeping bentgrass seeds (cv. Penncross, 4 g/m^2^) were sowed in containers (25 cm length, 15 cm width, and 10 cm height) filled with quartz sand and distilled water, and all containers were randomly placed in the growth chamber (21/18 °C (day/night), 65% relative humidity, and 750 μmol m^−2^·s^−1^ PAR) for 10 days of germination. Then, seedlings were cultivated with Hoagland’s solution [61] for another 20 days. For priming treatment, the 30-day-old plants were carefully moved out from Hoagland’s solution, and then transferred to new Hoagland’s solution containing 0.5 mmol/L GABA [14] or 2 mmol/L 3-MPA [62] (GABA or 3-MPA was dissolved in Hoagland’s solution) for 2 days. Untreated, GABA-pretreated, and 3-MPA-treated plants were then transferred into a new normal Hoagland’s solution without GABA and 3-MPA and placed in a normal growth chamber (as described above) or in a high-temperature growth chamber (38/33 °C (day/night), 65% relative humidity, and 750 μmol m^−2^·s^−1^ PAR) for 30 days. Hoagland’s solution was updated every day to avoid changes of solution concentration during normal cultivation and heat stress. All plant materials were completely placed in the growth chamber. A total of six treatments (C: control; C+GABA: control+GABA; C+3-MPA: control+3-MPA; H: heat; H+GABA: heat+GABA; H+3-MPA: heat+3-MPA) and four independent biological replicates per treatment were set. Leaves were collected for analyzing all parameters.

### 4.2. The Measurement of Endogenous GABA, Water Status, and Photosynthesis

The endogenous GABA content was determined by using the method of enzyme linked immunosorbent assay (ELISA). The Assay Kit was purchased from Shanghai Enzyme-linked Biotechnology Co., Ltd., China. Leaf EL is calculated as a percentage of C_initial_/C_max_ [63]. The 0.1g fresh leaves were immersed in 30 ml deionized water and shaken for 24 h. The initial conductivity (C_initial_) is measured by conductivity meter (YSI 32, yellow spring, OH). Then, leaves were autoclaved at 120 °C for 20 min to measure the maximum conductivity of the solution (C_max_). Leaf RWC was calculated using the formula RWC (%) = [(FW − DW)/(TW ‒ DW)] × 100% [64]. Fresh leaves were collected from plants and immediately weighed for fresh weight (FW), and then leaves were immerged in distilled water for 12 h. After being gently wiped dry, turgid weight (TW) was weigh. Leaves were then dried at 80 °C for 72 h to get a dry weight (DW). For Chl content in leaves, fresh leaves (0.1 g) were soaked in 10 ml 95% alcohol and 80% acetone mixture (1:1) for 48 h in darkness, and then leaf extracts were measured at 663 and 645 nm by spectrophotometer (Spectronic in Instruments, Rochester, New York, USA). The Chl content was calculated according to the formula described in Arnon [65]. The Fv/Fm and PIABS were measured using a Chl fluorescence system (Pocket PEA, Hansatech, the United Kingdom), and a layer of leaves was adapted to darkness for 30 min using leaf clips, and the Fv/Fm ratio and PIABS were recorded. Pn and WUE of 10 individual leaves per replicate per treatment were measured by using a portable photosynthesis system (CIRAS^−3^, PP Systems, Norfolk, United Kingdom).

### 4.3. The Measurement of Oxidative Damage and Total Antioxidant Capacity

For MDA, fresh leaves (0.2 g) were ground with 50 mM cold phosphate buffer (4 mL, pH 7.8) containing 1% (w/v) polyvinylpyrrolidone. The homogenate was centrifuged at 4 °C for 30 min at 12000 g. The extraction (0.5 mL) was mixed with the reaction solution (1 mL), which contained 20% (w/v) trichloroacetic acid and 0.5% (w/v) thiobarbituric acid. The mixture is heated for 15 min in a water bath at 95 °C and then cooled rapidly in an ice–water bath. The homogenate was centrifuged at 8000 g for 10 min. The absorbance of supernatant was measured at 532, 600 and 450 nm [66]. For O_2_^−^, 0.1 g of leaves was ground with 1.5 mL 65 mM PBS (pH 7.8) and centrifuged at 4 °C for 30 min at 10000 g to collect supernatant. Reaction mixture containing supernatant (0.5 mL), PBS (0.5 mL), and 10 mM hydrochloride (0.1 mL) was incubated in water bath (25 °C) for 20 min, and then 2 mL mixed solution (58 mM sulfonamide and 7 mM alpha-naphthylamine) was added to the reaction mixture for 20 min in 25 °C water bath. The chloroform (2 mL) was added to reaction mixture. Absorption was measured at 530 nm [67]. The content of H_2_O_2_ was determined as follows. Leaves (0.1 g) were homogenized with 5 mL 0.1% TCA and centrifuged for 20 min at 12000 g, and the supernatant (0.5 mL) mixed with 10 mM potassium phosphate (0.5 mL) and KI (1 mL). Oxidation products were measured at 390 nm [68]. The TAC was measured using the Assay Kit (Suzhou Comin Biotechnology Co., Ltd., China) according to manufacturer’s instructions.

### 4.4. Genes Expression Analyses

Genes transcription levels were detected by real-time quantitative polymerase chain reaction (qRT-PCR). Total RNA was extracted from 0.15 g fresh leaves by using Rneasy Mini Kit (Qiagen, Duesseldorf, Germany). The RNA was reverse-transcribed to the cDNA using a revert Aid First Stand cDNA Synthesis Kit (Fermentas, Lithuania). Primers of heat shock factor genes (*HSFA-6a*, *HSFA-2c*, and *HSFB-2b*), heat shock protein genes (*HSP17.8*, *HSP26.7*, *HSP70*, and *HSP90.1-b1*), and the gene encoding ascorbate peroxidase 3 (*APX3*) were used for qPCR (Table 1); *β-Actin* was used as the internal control. The PCR protocol conditions for all genes as follows; 5 min at 94 °C and 30 s at 95 °C (40 repeats of denaturation), annealing 45 s at 58–64 °C, and amplicon from 60 to 95°C. Transcript levels of all genes were calculated according to the formula 2^−∆∆Ct^ described by Xia et al. [69].

### 4.5. Statistical Analysis

The data was analyzed by using SPSS 20 (IBM, Armonk, NY, USA) and the SAS (SAS 9.1, SAS Institute, Cary, NC). Differences among treatments were tested by using Fisher’s protected least significance (LSD) test (Dunnett’s test) at a 0.05 probability level. The two-way ANOVA was made before using the Fisher’s LSD.

## 5. Conclusions

The mediation of endogenous GABA level could significantly affect heat tolerance in perennial creeping bentgrass. The GABA pretreatment is an effective way to improve heat tolerance of creeping bentgrass through effectively maintaining water balance, enhancing photosynthesis, and reducing oxidative damage caused by high temperature stress. On the contrary, the inhibition of GABA accumulation by the application of GABA biosynthesis inhibitor (3-MPA) further aggravates heat damage in leaves of creeping bentgrass. More importantly, the current results indicate that the GABA-regulated thermotolerance is associated with the activation and enhancement of HSF pathways in creeping bentgrass. The GABA significantly upregulates transcript levels of genes encoding heat shock factors (*HSFA-6a*, *HSFA-2c*, and *HSFB-2b*), heat shock proteins (*HSP17.8*, *HSP26.7*, *HSP70*, and *HSP90.1-b1*), and ascorbate peroxidase 3 (*APX3*), whereas the inhibition of GABA biosynthesis depressed these genes expression during heat stress. Further research is still needed about thermal stress signal transduction induced by GABA in plants.

## Figures and Tables

**Table 1:** Primer sequences and annealing temperature of analyzed genes.

Target Gene	Chromosome	Forward Primer (5’-3’)	Reverse Primer (5’-3’)	Tm (◦C)
*HSFA-2c*	3	GCCGTCCAATGTGCCTCCATC	CGCTTCAGCCTGTCAATCTCTTCC	62
*HSFA-6a*	1	CACCTTCGAGGAGCTGGCATTG	TGTCTATCTCCGCCTGCTCATCC	62
*HSFB-2b*	8	GCTGGAGAACTCACGGCTAACG	CTGCTGAGTGTCGCTGTACTTGG	62
*HSP17.8*	5	GGCGACATCAAGGTGCAGGTG	ACTTGGCGTCCTCCTTCTCCTC	62
*HSP26.7*	4	GCGGCGAGCACAAGAAGGAG	GACCTGCACGTCGATGACCTTG	64
*HSP70*	2	GCTGAGGATGACGAAGACGAAGAC	ATCGGCGTTGACGTGAACCATC	60
*HSP90.1-b1*	5	ACGAAGTGATCTTCATGGTGGATG	CTGTCGCCGAGAATGTCCTTGAT	64
*APX3*	3	GGCTCAAGATCGCAGTCG	TGGTAGAGGTCGGCATACG	58
*β-Actin*	5	CCTTTTCCAGCCATCTTTCA	GAGGTCCTTCCTGATATCCA	58
